# Alterations in serum levels of trace element in patients with breast cancer before and after chemotherapy

**DOI:** 10.22088/cjim.9.2.134

**Published:** 2018

**Authors:** Norjis Ahmadi, Soleiman Mahjoub, Reza Haji Hosseini, Mostafa TaherKhani, Dariush Moslemi

**Affiliations:** 1 Department of Biochemistry, Faculty of Sciences, University of Payam Noor Tehran, Tehran, Iran.; 2 Cellular and Molecular Biology Research Center, Health Research Institute, Babol University of Medical Sciences, Babol, Iran.; 3 Department of Clinical Biochemistry, School of Medicine, Babol University of Medical Sciences, Babol, Iran.; 4 Student Research Committee, School of Medicine, Babol University of Medical Sciences, Babol, Iran.; 5 Department of Nursing, Takestan Medical Sciences Branch, Islamic Azad University, Takestan, Iran. -; 6 Cancer Research Center, Health Research Institute, Babol University of Medical Sciences, Babol, Iran.

**Keywords:** Breast cancer, Chemotherapy, Trace elements, Adriamycin, Cytoxan

## Abstract

**Background:**

Breast cancer is the most common serious disease around the world. The trace elements have a vital role in the metabolism and chemotherapy may change the level of metal ions. Due to the ambiguity of the existence in this regard, the study examined the trace element serum levels in women with breast cancer before and after chemotherapy .

**Methods:**

Sixty patients were studied undergoing specialist. First sampling was taken before chemotherapy (after 4 weeks of surgery) and second sampling was taken after the completion of 3 courses of chemotherapy, approximately 9 weeks after the first chemotherapy. The patients took Adriamycin 60mg/m^2^ Cytoxan 600mg/m^2^. Serum zinc and iron levels were measured using standard spectrophotometric method. Measurement of serum copper was done by atomic absorption spectroscopy*.*

**Results:**

Serum zinc and iron levels in women after chemotherapy significantly decreased (p<0.001), however, the serum level of copper increased but was not significant (P=0.676).

**Conclusions:**

Our findings demonstrate significant decrease in zinc and iron levels in breast cancer patients after 3 courses of Adriamycin and Cytoxan chemotherapy. Prescribing zinc supplements can be useful after chemotherapy.

Breast cancer is one of the most common diseases in Western countries including the United States of America (1). In the US in 2012, breast cancer was considered of the 29% of the newly diagnosed cancers. One of 8 women is diagnosed with breast cancer, and annually about 40000 people die from breast cancer. Definitely, men can also be diagnosed with breast cancer, but it is less than women and is also less dangerous in men (2). In Iran, in every 10 to 15 people, one person is diagnosed with breast cancer (3). A combined therapy such as surgery, chemotherapy, radiotherapy, hormone therapy and immunotherapy may usually be used for the treatment of this cancer (4). Today, chemotherapy is considered as one of the major treatments of cancers. In this method, a variety of anticancer drugs are used alone or in combination with other components (5). There are different types of chemotherapy in the treatment of breast cancer. One of the most important methods is chemotherapy with Adriamycin and Cyclophosphamide (AC) that has some advantages and disadvantages (6). Adriamycin (Doxorubicin) is an active chemotherapeutic agent in the treatment of human neoplast. Adriamycin is a chemical compound that prevents creating DNA and DNA repair enzymes. Cells cannot live without DNA and will die. The major problem of this drug is that it cannot distinguish cancer cells from the healthy ones, but has also more adverse effects on the cancer cells, because cancer cells divide and group quickly. Cyclophosphamide (cytoxan) is a chemical compound that stops cancer cells replication.

Cyclophosphamide is dose-dependent. At higher doses, it is associated with increased cytotoxicity and immunosuppression, while at low continuous doses, it shows immunostimulatory and antiangiogenic properties. So the combination of these two drugs is more effective in destroying cancer cells (7). The trace elements and some metals have a significant and vital role in metabolism. Zinc is a vital trace element in many homeostatic mechanisms of the body. More than 200 zinc enzymes are known. Zinc activates some enzymes of protein metabolism, and is important for immune function. Zinc acts as a cofactor in some antioxidant enzymes such as dehydrogenase and superoxide dismutase (SOD). A number of zinc enzymes require two or more metal ions for full activity, such as Cu–Zn superoxide dismutase. SOD is an important antioxidant defense in nearly all living cells exposed to oxygen (8, 9). 

Antioxidant system can be changed and disturbed in the course of chemotherapy. Taherkhani et al. (2017) reported that chemotherapy with adriamycin and cytoxan can significantly increase oxidative stress and decrease total antioxidant capacity in women with breast cancer (10). Free radicals are neutralized by some antioxidant enzymes such as glutathione peroxidase, catalase and SOD. Copper and zinc are necessary for optimal performance of SOD. The process of oxidative stress mostly occurs in an imbalance of the concentration of trace elements which is used in the structure of antioxidant enzymes (11). Generally, oxidative damages are often associated for reducing function of enzymatic antioxidant, and changes of some trace elements can affect the activity of antioxidant enzymes (12, 13).

Metal ion chelating agents including transferrin, ferritin, ceruloplasmin, albumin and small molecules including vitamins, act as cell redox balance (14). Iron, an essential metal, acts as a catalyst for the production of free oxygen species. In patients with breast cancer, estrogen of blood circulation facilitates releasing iron for ferritin storage. Iron stimulates oxidative stress in the breast tissue. Similarly, copper produces free species of oxygen through activation of several structural peroxidases. The free radicals stimulate mutations by damaging DNA. Therefore, an increase in serum levels of iron and copper as compound agents may cause breast cancer. There are significant and statistically considerable differences in normal distribution of some trace elements (copper and zinc) in patients with various cancers (15). Increasing iron and copper levels through establishing reactions of Haber Weiss and Fenton can lead to free radicals and mutagenic effects (16). Zinc, as an antioxidant, acts as a protecting factor against cancer in cell cycle. Zinc is also essential for activity of man’s transcription factors and for proteins that recognize specific DNA sequences and regulate gene transcription (17). 

Furthermore, zinc can directly prevent the development of DNA gaps and gene mutation and thereby leads to reducing the risk of cancer (18). Therefore, reduction in serum zinc level can lead to various cancers by decreasing protecting effects and increasing antioxidant effects. The resultant cellular function reduces the number of tumor cells and tumor size (19). Estimation of the serum levels of metal ions has a potential role in monitoring of patients with breast cancer (20). Due to ambiguities in this regard, the present study investigated some trace elements before and after three cycles of Adriamycin and Cytoxan (AC) chemotherapy in the serum of women with breast cancer. 

## Methods

**Study design: **A total of 60 breast cancer patients treated with chemotherapy after surgery were entered into the study under the supervision of an oncologist. After obtaining consent from the patients, their blood samples were obtained for routine blood sampling tests in a fasting state. Blood samples were taken from the patients before the treatment and after receiving three cycles of chemotherapy with AC regimen. After clotting, the blood samples were centrifuged for 15 min with speed of 3000 rpm. Serum samples were kept in capped Eppendorf microtubes in -80°C, at the Biochemistry Research Laboratory of Babol University of Medical Sciences until the end of sampling. The first sampling was done before chemotherapy (4 weeks after surgery), and the second sampling after three courses of chemotherapy (that is usually 9 weeks after the first chemotherapy). 

All medicines were allowed to be used in Iran and were prepared largely from Italy and France with international standards. The drugs prescribed (Adrimaycin 60 mg/m^2^, Cytoxan 600mg/m^2^) were injected intravenously every 3 weeks. Inclusion criteria were: patients with breast cancer aged 30-60 who had chemotherapy after surgery plus all patients with ductal stages II - III carcinoma. Exclusion criteria were: diabetes, inflammatory disease, taking supplementary vitamins and antioxidants plus patients who had chemotherapy before, were excluded from the study. The number of patients in different stages is as follows: in stage II (36 patients) and in stage III (24 patients). Copper, zinc, iron and BMI were measured and compared to patients with breast cancer before and after chemotherapy.

**Laboratory methods: **Copper serum levels were measured by atomic absorption spectrophotometer equipped with graphite furnace. Standard solutions were prepared using copper sulfate and nitric acid 0.1%. The standard solutions were diluted sequentially in different concentrations on the basis of ppb. 10 ml standard solution was injected with different concentrations (10, 20, 40, 80 ppb) to atomic absorption spectrometer and the standard curve was drawn. Concentration of samples was measured by device and results were reported according to µg/dl. Zinc concentration in serum samples of the patients was determined by a commercial kit (Biorex Diagnostics Ltd, UK). Specifically, zinc compounds with chromogenic 5-Br-PAPS is a stable color complex in which the color intensity is proportional to the amount of zinc of the sample. Precision of the kit for intra -assay (within run) was CV=0.91% and for inter-assay (between run) was CV=2.97 %. Absorption of samples and standard were read in wavelength 546 nm against blank and reported on the basis of µg/dl. 

Iron concentration in serum samples was determined by Ferrocene method with a commercial kit (Darman Faraz Kav Co., Iran) using a UV-Vis spectrometer. Iron attached to proteins in acidic buffer (pH 5.4) was released while proteins do not precipitate. The free iron is reduced and then produces a purple complex by combining with ferrocin. Intensity of the produced color depends on the concentration of serum iron. Hence, absorption of test and standard tubes against blank tubes in wavelength 562 nm was reported. Sample iron concentration was calculated in micrograms per deciliter. Demographic data including age, height and weight of all patients were recorded. Body mass index (BMI) was calculated using the following formula:

BMI (kg/m²) = Weight(kg) / Height(m²)

Data obtained from experiments were analyzed using statistical software SPSS Version 19 and paired t-test was used to compare the mean values of variables in patients with breast cancer before and after chemotherapy. A p<0.05 was considered significant. In addition, independent t-test was used to compare mean values of the variables based independently on age and stage in patients with breast cancer before and after chemotherapy. 

## Results

There was a significant decrease in the average concentration of Zn in serum of women with breast cancer after chemotherapy compared with previous chemotherapy (p<0.001). Changes in the concentration of Fe in the serum of patients were same with Zn and showed significant decrease after chemotherapy (p<0.001).

There was an increase in the average concentration of Cu in the serum of patients after chemotherapy rather than before chemotherapy, but it was not significant (P=0.676). Also, the mean of BMI index of the patients after chemotherapy increased but it was not significant (P=0.336) (table 1).

**Table 1:** Serum levels of metal ions and BMI before and after chemotherapy in women with breast cancer.

**Chemotherapy** **Variables**	**Before** **Mean±SD**	**After** **Mean±SD**	**P-value**
Zn (µg/dL)	74.96±9.99	62.89± 9.01	0.001
Fe (µg/dL)	20.84± 79.29	17.31±54.68	0.001
CU (µg/dL)	32.8± 135.75	31.5± 137.99	0.676
BMI (Kg/m^2^)	3.79± 27.94	3.74± 27.99	0.336

The changes in metal ions concentration and BMI in patients with breast cancer before and after chemotherapy in different clinical stages of disease were shown in table 2. 

**Table 2:** Metal ions concentration and BMI in women with breast cancer before and after chemotherapy in different stages

**Variables**	**Chemotherapy ** **groups**	**Stage II** **(n=36)**	**Stage III** **(n=24)**	**P-value**
CU(µg/dL)	Before	138.72	131.28	0.394
	After	148.72	121.89	0.001*
	P-value	0.188	0.184	-
Zn(µg/dL)	Before	75.03	74.85	0.945
	After	63.54	61.92	0.502
	P-value	0.001*	0.001*	-
Fe(µg/dL)	Before	82.8	74.02	0.111
	After	51.63	59.25	0.095
	P-value	0.001*	0.005*	-
BMI (Kg/m^2^)	Before	4.5±27.28	4.9±28.93	0.09
	After	4.6±27.34	4.7±29.04	0.08
	P-value	0.326	0.307	-

Table 3 shows the metal ions concentration and BMI in patients with breast cancer before and after based on age.

**Table 3:** Changes of metal ions concentration and BMI in women with breast cancer before and after chemotherapy in different ages

**Variables**	**Chemotherapy ** **Groups**	**48> age** **(n=34)**	**age ≥48** **(n=26)**	**Pvalue**
CU(µg/dL)	Before	136.83	134.12	0.248
	After	140.67	133.96	0.235
	P-value	0.552	0.98	-
Zn(µg/dL)	Before	74.47	75.69	0.979
	After	63.43	62.09	0.787
	P-value	0.001	0.001	-
Fe(µg/dL)	Before	77.98	81.26	0.450
	After	59.22	47.86	0.490
	P-value	0.001	0.001	-
BMI (Kg/m^2^)	Before	4.7±28.40	4.6±27.33	0.262
	After	4.4±28.50	4.5±27.34	0.205
	P-value	0.115	0.841	-

## Discussion

In the present study, alterations of serum levels of some metal ions before and after 3 courses of adriamycin and cytoxan (AC) chemotherapy were investigated in women with breast cancer. In many cases of breast cancer, a combination of two or more drugs were used as chemotherapy treatment. This depends on the breast cancer subtypes, stage of the disease, status of hormone receptors, age and so on. The available therapeutic methods in the treatment of breast cancer include surgery, chemotherapy, hormone therapy and radiotherapy.

 Chemotherapy is usually used after surgery for killing any remaining cancer cells in the patient (21). Depending on the type of drug usege and dosage, chemotherapy causes the increase in production of free radicals and finally leads to oxidative stress (10). 

Anthracyclines by producing superoxide and hydrogen peroxide and iron-mediated generation of free oxygen radicals can damage DNA, proteins and cell membranes. They inhibit DNA synthesis by intercalating between base pairs of the DNA chains, therefore preventing the replication of rapidly growing cancer cells (22). Anthracyclines such as Adriamycin (Doxorubicin) can cause cardiotoxicity (23, 24). There exists an evidence that the effect of cardiotoxicity increases in long-term survivors, from 2% after 2 years to 5% after 15 years (25).

In our study, the serum level of zinc decreased after 3 courses of injections of the chemotherapy support drugs. This finding is compatible with the results of *Faber et al.* (26). In the present study, significant reduction in the level of zinc after chemotherapy led to reducing protective effects against oxidative stress in patients. Zinc as an antioxidant role in cell cycle acts as protection element against carcinogenesis. Zinc is also vital for functions of many transcription factors and proteins that recognize certain DNA sequences and regulate gene transcription, so zinc deficiency can cause induced oxidative stress (27). Copper-zinc superoxide dismutase is an antioxidant enzyme present in the nucleus, cytosol, peroxisomes, and mitochondrial inter membrane space of human cells. The enzyme function is lowering the steady-state concentration of superoxide (28). According to our study, the concentration of serum iron was significantly reduced after 3 courses of the AC chemotherapy. Iron is an important metal required for a number of essential cell functions. Iron is necessary for hemoglobin and myoglobin biosynthesis and hence is a vital nutrient. However, iron can also be dangerous as a catalyst of free radical reactions. Accordingly, intracellular iron homeostasis and body iron balance are tightly regulated (29). 

In a study on peripheral arterial disease, Zocharsky et al. observed that reduction of iron levels was associated with reducing risk of cancer and death, these findings are compatible with our results (30). One of the reasons for the decreased serum levels of iron in patients with breast cancer can be caused by long-term chronic bleeding and hematuria that eventually leads to the creation of anemia (31). 

In the current study, the amount of serum copper increased a little after chemotherapy but was not significant. Similarly, in one study that used Adriamycin for chemotherapy of cancer patients, the copper level did not significantly change after chemotherapy (28). Interaction of Cu with hydrogen peroxide generates more reactive oxygen species, such as hydroxyl radicals. These reactive oxygen species have been considered as being responsible for the process of carcinogenesis (32). We also studied BMI index and the serum levels of Zn, Cu, and iron based on the stage of disease and age of the patients. According to our finding, BMI and the serum levels of these metal ions did not significantly change based on stage of disease and age. The limitation of the present study was the investigation of the metal ions before and after 3 courses of chemotherapy, approximately 9 weeks after the first chemotherapy. Full chemotherapy medicines for the patients were adriamycin 60mg/m^2^, cytoxan 600mg/m^2^, Taxoter 80mg/m^2^**, **but in 3 courses of chemotherapy, they received only adriamycin and cytoxan (AC), as a result, our study only focused on the effects of AC chemotherapy in breast cancer patients. Trace elements are known to play a vital role in metabolism. The measurement of trace elements and metal ions are valuable in cancer patients. For further studies, we suggest the measurement of other trace elements after chemotherapy in breast cancer patients and also the investigation on the effects of different chemotherapy regimens. 

In conclusion, our findings demonstrate significant decrease in serum zinc and iron levels in breast cancer patients after 3 courses of Adriamycin and Cytoxan chemotherapy. Prescribing antioxidant and zinc supplements and nutritional advice for cancer patients can be useful after chemotherapy.
